# Surgeons' Perspective, Learning Curve, Motivation, and Obstacles of Full-Endoscopic Spine Surgery in Thailand: Results From A Nationwide Survey

**DOI:** 10.1155/2022/4971844

**Published:** 2022-03-11

**Authors:** Vit Kotheeranurak, Wongthawat Liawrungrueang, Verapan Kuansongtham, Pornpavit Sriphirom, Narongsak Bamrungthin, Gun Keorochana, Pritsanai Pruttikul, Worawat Limthongkul, Weerasak Singhatanadgige, Suthipas Pongmanee, Rattalerk Arunakul, Monchai Ruangchainikom, Phanunan Sasiprapha, Roongrath Chitragarn, Saran Pairuchvej, Teerachat Tanasansomboon, Khanathip Jitpakdee

**Affiliations:** ^1^Department of Orthopedics, Queen Savang Vadhana Memorial Hospital, Sriracha, Chonburi, Thailand; ^2^Spinal Unit, Orthopedic Department, Chiang Mai University, Chiang Mai, Thailand; ^3^Spine Institute, Bumrungrad International Hospital, Bangkok, Thailand; ^4^Department of Orthopedics, Faculty of Medicine, Rangsit University, Pathum Thani, Thailand; ^5^Pakchong Nana Hospital, Pak Chong, Nakhon Ratchasima, Thailand; ^6^Orthopedic Department, Faculty of Medicine Ramathibodi Hospital, Mahidol University, Bangkok, Thailand; ^7^Department of Orthopedics, Lerdsin hospital, Bangkok, Thailand; ^8^Department of Orthopaedics, Faculty of Medicine, Chulalongkorn University, Bangkok, Thailand; ^9^Center of Excellence in Biomechanics and Innovative Spine Surgery, Chulalongkorn University, Bangkok, Thailand; ^10^Department of Orthopaedics, Faculty of Medicine, Thammasat University, Thailand; ^11^Department of Orthopaedic Surgery, Faculty of Medicine Siriraj Hospital, Mahidol University, Bangkok, Thailand; ^12^Orthopedic department, Udon Thani Hospital, Udon Thani, Thailand; ^13^Department of Orthopaedics, Phramongkutklao Hospital and College of Medicine, Bangkok, Thailand; ^14^Orthopedic department, Samutsakhon Hospital, Samutsakorn, Thailand

## Abstract

**Objective:**

To report a nationwide survey of the endoscopic spine surgeons across Thailand. Furthermore, the survey will be focused on the perspective of experience, learning curve, motivations, and obstacles at the beginning of their practices.

**Materials and Methods:**

The online survey consisting of 16 items was distributed to spine surgeons who are performing endoscopic spine surgery in Thailand via the Google forms web-based questionnaire to investigate participants' demographics, backgrounds, experience in endoscopic spine surgery, motivations, obstacles, and future perspectives. The data was recorded from January 7, 2020 to January 21, 2022. Descriptive statistics were used for analysis.

**Results:**

A total of 42 surveys were submitted by 6 neurosurgeons (14.3%) and 36 orthopedic surgeons (85.7%). From the surgeons' perspective, the average number of cases that should be performed until one feels confident, consistently good outcomes, and has minimal complications was 27.44 ± 32.46 cases. For surgeons who starting the endoscopic spine practice, at least 3 workshop participation is needed. Personal interest (39 selected responses) and trending marketing or business purpose (25 selected responses) were the primary motivators for endoscopic spine surgery implementation. Lack of support (18 selected responses) and afraid of complications (16 selected responses) were pertinent obstacles to endoscopic spine surgery implementation.

**Conclusions:**

The trend of endoscopic spine surgery has continued to grow in Thailand, shown by the rate of implementation of endoscopic spine surgery reported by Thai spine surgeons. The number of appropriate cases until one feels confident was around 28 cases. The primary motivator and obstacles were personal interest and lack of support.

## 1. Introduction

Endoscopic spine surgery is being used to treat lumbar spine diseases across the world [1]. Current evidence shows that full-endoscopic spine surgery is comparable to other minimally invasive spine surgery (MISS) or open spine surgery [2–5]. Full-endoscopic spine surgery is a common surgical procedure for treating lumbar disc herniation (LDH). Different types of endoscopic discectomy have been introduced and evolved since 1980, including transforaminal endoscopic lumbar discectomy (TELD) and interlaminar endoscopic lumbar discectomy (IELD) [4–6]. Specifically, when compared to more traditional MISS techniques, several randomized-controlled trials and meta-analyses have shown that patients undergoing spinal endoscopy for the treatment of lumbar stenosis, lumbar disc herniations, and cervical radiculopathy have similar outcomes [2–5].

This shifting trend, from more open to less invasive surgical techniques, is similar to what has happened in the past with laparoscopy and joint arthroscopy, where the patient would opt for less morbid operations and surgeon efforts to speed up postoperative recovery. However, the learning curve for spinal endoscopy is substantial or some would even mention the endoscopic spine surgery as having a steep learning curve, even for experienced surgeons. The learning curve greatly varies depending on the treatment being performed [1, 7].

The aim of this study was to firstly report a nationwide survey of the endoscopic spine surgeons across Thailand. Furthermore, the survey will be focused on the perspective of a learning curve, motivations, and obstacles at the beginning of their practices.

## 2. Materials and Methods

The online survey was devised by the authors who are key endoscopic spine surgeons to collect responses from spine surgeons inquiring about their perspectives in endoscopic spine surgery. The nationwide online questionnaire was developed in the English language in the Google Forms (Mountain View, California, USA) and was distributed to spine surgeons in different regions of Thailand via a link through social networks including Line group, Facebook, and WhatsApp. The survey could be accessed by computer, laptop, or smartphone and was available to be completed online within 2 weeks of the response period.

The questionnaire consisted of 16 items in form of single short answer questions, multiple-choice questions, and checkboxes for multiple answers. The proposes of this survey were to investigate participants' demographics, backgrounds, experience in endoscopic spine surgery, their motivations and obstacles at the beginning of the practice, and future perspectives in their opinions.

Participants were asked to answer the following questions:

(1) Demographic data
  - Sex
  - Age
  - Region of workplace in Thailand
  - Postgraduate residency training
(2) Experience in endoscopic spine surgery
  - Number of completed cases performed with uniportal approach
  - Number of completed cases performed with biportal approach
  - Proportion of approach (interlaminar vs transforaminal)
  - Proportion of disease (discectomy vs decompression)
  - Percentage of practice devoted to endoscopic spine surgery
  - Years of experience performing endoscopic spine surgery
(3) The beginning of endoscopic spine surgery, according to the participant's opinion
  (i) Number of workshop attendances needed to be completed before starting endoscopic spine surgery
  (ii) Number of cases needed to be performed to be confident, have minimal complications, and result in good outcomes
  (iii) Motivations to start endoscopic spine surgery
    - Personal interest
    - Trending/marketing/business purpose
    - Patient demand
    - Pressure by organization
  (iv) Obstacles at the beginning of the practice
    - No obstacles
    - Lack of support
    - Financial problem
    - Too long operative time
    - Afraid of potential complications
    - Doubt about advantages over conventional techniques
(4) Future perspective on endoscopic spine surgery, according to the participant's opinion
  (i) Qualification system for spine surgeons prior to performing endoscopic spine surgery
    - Mandatory
    - Unnecessary
    - Not sure
    - Impossible to implicate
  (ii) Perspective on endoscopic spine surgery in the future
    - Gold standard
    - Alternative
    - Fade away

An invitation link to the online survey was sent to participants on January 7, 2020 and responses were recorded until January 21, 2022. All participants were required to answer all the items to ensure a complete data collection without missing parameters. The responses were collected anonymously on the Google Forms to blind the authors to the identity of the participants. The time-stamp of each questionnaire was also recorded at the end of the survey, when participants submitted the responses. Once all the surveys were completed, all responses were retrieved in the form of Microsoft Excel format (.xlsx) which was imported into the statistical analysis software, IBM SPSS (version 25).

Descriptive statistics was performed to count responses, determine the demographic characteristics, and calculate the mean, median, range, percentages, standard deviation (SD), and interquartile range (IQR). Categorical data were presented as number of cases and percentages. Continuous data were presented as mean with SD or median with IQR, depending on the data distribution. The multiple-choice items or checkboxes were illustrated with graph, bar, and pie charts. Statistical significance was considered when the *P* value was 0.05 or less, and a confidence interval of 95% was considered for all statistical tests. Linear regression and kappa analysis of agreement were performed to measure the consistency of the responses.

## 3. Results

**Figure 1:**
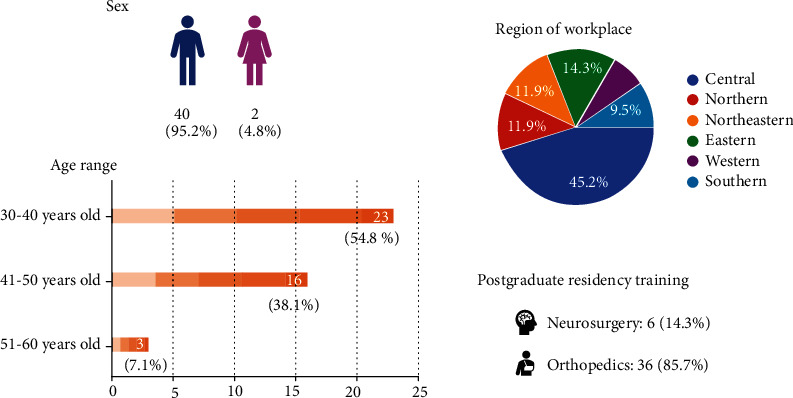
Demographics of participants in this study.

Spine surgeons who are currently performing endoscopic spine surgery in Thailand were invited to participate in an online survey using a Google form web-based application. Forty-two surgeons completed the survey. Male surgeons accounted for the great majority of responders 40 (95.2%), with female surgeons accounting for only 2 (4.8%). There were 36 orthopedic surgeons (85.7%) and 6 neurosurgeons (14.3%). Nearly half of the participants worked in Central Region 19 (45.2%), Eastern Region 6 (14.3%), Northeast Region 5 (11.9%), North Region 5 (11.9%), Southern Region 4 (9.5%), and Western Region 3 (7.1%). From the survey, surgeons aged 30-40 years were represented by 23 (54.8%), surgeons aged 41-50 years by 16 (38.1%), and surgeons aged more than 50 years by 3 (7.1%). There were no surgeons under the age of 30 or more than 60 who participated in the survey. As illustrated in Figure 1, the data obtained for the surgeons includes demographic information as well as their baseline date.

### 3.1. Uniportal Endoscopic Spine Surgery Experience

There were 24 surgeons with less than 50 cases experience, 6 surgeons with 50-100 cases experience, 7 surgeons with 100-500 cases experience, and 5 surgeons with more than 500 cases.

### 3.2. Biportal Endoscopic Spine Surgery Experience

Online responses showed that 34 surgeons have performed less than 20 biportal surgeries, 4 surgeons have performed 30-50 cases, 2 surgeons have performed 51-100 cases, and only 2 surgeons have performed more than 100 cases.

### 3.3. Endoscopic Approach

There were 7 surgeons who preferred a 100% interlaminar approach, 24 surgeons who preferred a 90% interlaminar: 10% transforaminal approach, 9 surgeons who preferred a 75% interlaminar: 25% transforaminal approach, 1 surgeon who preferred a 50% interlaminar: 50% transforaminal approach, and 1 surgeon who preferred a 25% interlaminar: 75% transforaminal approach. None of them responded with a 100% transforaminal approach. The approach proportion is shown in Table 1.

### 3.4. Diseases

There were 4 surgeons who have performed 100% discectomy, 13 surgeons who have performed a 90% discectomy: 10% decompression, 13 surgeons who have performed a 75% discectomy: 25% decompression, 7 surgeons who have performed a 50% discectomy: 50% decompression, 2 surgeons who have performed a 25% discectomy: 75% decompression, 1 surgeon who has performed a 10% discectomy: 90% decompression. None of the respondents reported a 100% decompression surgery. The disease proportion is shown in Table 2.

### 3.5. Endoscopic Spine Surgery Devotion in Practice and Years of Endoscopic Experience

In endoscopic spine surgery devotion, less than 25% were responded by 18 surgeons, 26% to 50% by 13 surgeons, 51% to 75% by 7 surgeons, and more than 75% by 4 surgeons.

For the years of endoscopic spine surgery practice, five surgeons selected “less than 1 year,” 24 surgeons selected “1-4 years,” 8 surgeons selected “5-10 years,” and five surgeons reported “more than 10 years,” shown in Table 3.

### 3.6. Perspective on the Workshop Attendance prior to Starting the Practice

**Figure 2:**
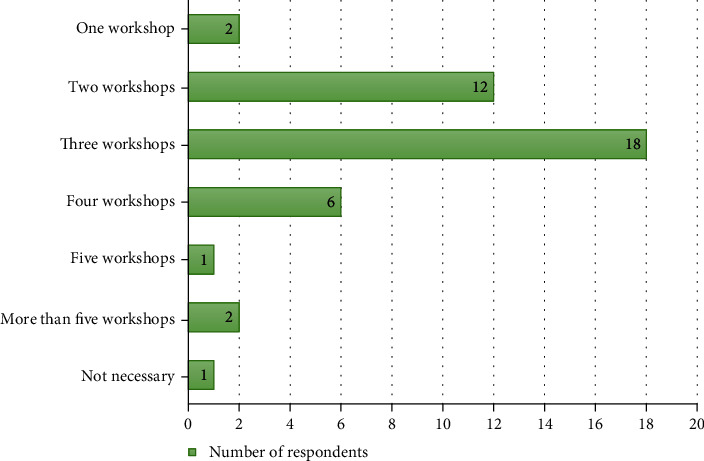
The number of workshops attendance needed before starting the endoscopic spine surgery.

The number of workshop attendance required before starting the endoscopic practice based on respondents' perspectives showed that “3 workshop requirements” were selected the most by 18 of the respondents (42.3%), “1 workshop requirement” was selected by 2 respondents (4.8%), “2 workshop requirements” were selected by 12 respondents (28.6%), “4 workshop requirements” were selected by 6 respondents (14.3%), “5 workshop requirements” were selected by 1 respondent (2.4%), “more than 5 workshop requirements” were selected by 2 respondents (4.8%), and “no workshop requirement” was selected by 1 respondent (2.4%), respectively (Figure 2).

### 3.7. Learning Curve (Based on Surgeons' Experience)

The average number of cases that should be performed until one feels confident, resulted in consistently good outcomes, and have minimal complications was 27.44 ± 32.46 cases from this survey result.

### 3.8. Motivation before Practicing Endoscopic Spine Surgery

**Figure 3:**
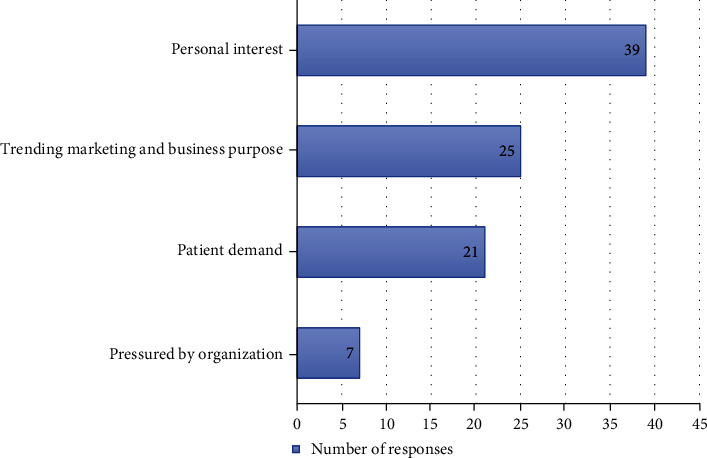
Motivation in endoscopic spine surgery.

The most selected motivation for endoscopic spine surgery was “personal interest” (39 selected responses), followed by “trending marketing and business purpose” (25 selected responses), “patient demand” (21 selected responses), and “pressured by organization” (7 selected responses). The motivation for endoscopic spine surgery is shown in Figure 3.

### 3.9. Beginner's Challenge and Obstacles

**Figure 4:**
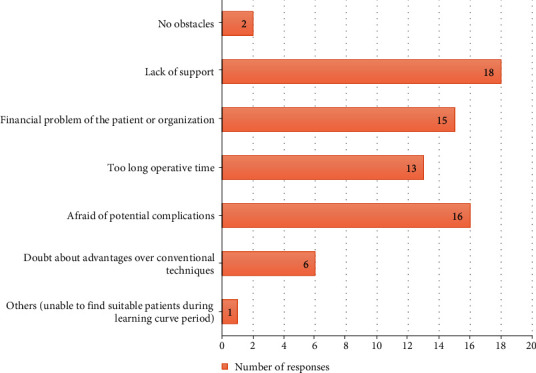
Beginner's challenge and obstacles.

When asked about the obstacles and challenges at the begging of the respondents' experience, the most selected responses were “lack of support” (18 selected responses), followed by “afraid of potential complications” (16 selected responses), “financial problem of the patient or organization” (15 selected responses), “too long operative time” (13 selected responses), “doubt about advantages over conventional techniques” (6 selected responses), and “unable to find suitable patients during the learning curve period” (1 selected responses), and 2 surgeons reported no obstacles at the beginning of their practices. The obstacles and challenges at the begging of the respondents' experience were shown in Figure 4.

### 3.10. Qualification System

According to the surgeons' responses, more than half of the respondents (25/42; 59.5%) reflected that a proper qualification system prior to starting endoscopic spine surgery practice is mandatory. Twelve surgeons (28.6%) selected “not sure,” three surgeons (7.1%) believed that the qualification system is not necessary, and two surgeons (4.8%) expressed the “impossible to implicate” response.

### 3.11. Perspective on Endoscopic Spine Surgery in the Future

When asked about the perspective of the upcoming state of endoscopic spine surgery, more than half of the respondents believed that the endoscopic spine surgery would become a gold standard in treating lumbar disc herniation, with the 22 votes (52.4%), and the rest of them (20 respondents, 47.6%) anticipated the endoscopic spine surgery will be just an alternative treatment.

## 4. Discussion

To the best of our knowledge, this study is the first Thai nationwide survey to investigate the current surgeon's perspectives on endoscopic spine surgery in many aspects that may reflect the current status of endoscopic spine surgery in Thailand and its fate in the future.

Participants' demographics revealed that more than half of surgeons who performed endoscopic spine surgery were in the early middle age as this surgical technique became increasingly popular when they graduated a spine fellowship training. Most of the surgeons worked in central region of Thailand which possibly due to more numbers of spine surgeons and higher patients' socioeconomic status in this area where the cost of endoscopic instrument was affordable. Most of the participants graduated from orthopedic residency training which was potentially a sampling bias due to most of the authors were orthopedists.

### 4.1. Experience with Performing Endoscopic Spine Surgery

Most of the participants were relatively new to endoscopic spine surgery as they mostly have less than 4 years of experience with devotion to this technique for less than 25% of their spinal surgeries. This reflects that the endoscopic spine surgery is starting to gain traction in Thailand as many surgeons are in the learning curve period.

From the survey, the interlaminar approach was preferred by most Thai surgeons, even though transforaminal access is the most approach performed worldwide. Compared with transforaminal approach, surgeons would be more familiar with anatomic visualization when they perform an interlaminar approach as when they do conventional open discectomy. The foraminal/extraforaminal disc herniation that warrants transforaminal approach is also relatively rare.

Most respondents also had more experiences in performing endoscopic discectomy for lumbar disc herniation than endoscopic decompression for spinal canal stenosis. The decompression procedure is likely to have more complexity involving more ligamentum flavum resection, more bone work, or need of over-the-top technique that technical demands and learning curves are needed. Additionally, in some circumstances, surgeons would prefer fusion procedure instead of decompression alone to treat spinal canal stenosis, as some controversies exist in this area [8, 9], unlike lumbar disc herniation which discectomy is usually indicated [10].

### 4.2. The Learning Curves

Participants considered the average required number of their performed cases for independent endoscopic spine surgery to be as high as approximately 30 cases with 3 workshops required until they felt comfortable with this technique to have satisfactory outcomes. This response was in accordance with previous literatures regarding learning curve of endoscopic spine surgery. Yang et al. reported the learning curve of endoscopic transforaminal decompression for lumbar spinal stenosis that the steady state for surgeons to perform this technique was achieved after approximately 35 cases when the operative time decreased significantly [11]. Hsu et al. also reported the learning curve of full-endoscopic discectomy that most surgeons approximately need a practice period of 3 years and 33 cases receiving the transforaminal procedure before they felt comfortable and achieved satisfactory results [12]. Our survey result in addition to previous studies may provide guidance for surgeons who intend to start performing endoscopic spine surgery. Regarding the perspective on workshop attendance prior to starting the practice, some surgeons answered that 1 or 2 workshops are sufficient for practice and is somewhat apprehensive. Thus, qualification system, ongoing, and monitored training should be emphasized.

### 4.3. Motivations and Obstacles at the Beginning

Participants were motivated to start endoscopic spine surgery by different reasons, mostly by their personal interest. Recent advancement of the procedure and evidences of its efficacy and outcomes may interest surgeons to start the practice. Trending marketing, business purpose, and patient demand are also considered as their motivations. Similar to other fields of surgery, trends of spinal operation continue to move towards minimally invasive approaches that allow patients less postoperative pain and faster return to activities and these advantages consequently become an appealing aspect to patients and also their doctors who need to catch up to meet these expectations. Moreover, some participants even responded that they were pressured by their organization to start the endoscopic surgery.

One of the most important questions in this survey is about their hindrances at the beginning of the endoscopic spine surgery. Most surgeons lacked support in practicing the procedure which possibly led to lack of confidence and fear of potential complications or unsatisfactory outcomes. This reflects that more workshops and support from the expert endoscopic spine surgeon are necessary to practically boost their confidence to do the operation with effectiveness and safety. Financial problem is also a major obstacle for both patients and organization to afford the high cost of instrumentation. A cost-effectiveness study of endoscopic spine surgery may be beneficial for organization to consider the healthcare cost associated with endoscopic procedure compared to the conventional technique [2]. Some participants considered that endoscopic spine surgery is taking too long operative time. This belief may be different among surgeons owing to their experience, preference, and learning curve of each procedure. However, previous literatures demonstrated some controversies in the comparison of operative time of conventional open and endoscopic technique [12, 13].

Despite evidences of many benefits from endoscopic spine surgery, some surgeons still hesitated to begin the practice because they were doubtful about advantages over the conventional techniques. According to the survey, this perception was mostly responded in senior spine surgeons who may have high experience of achieving consistently good results from conventional procedures.

### 4.4. Agreement of Qualification System and Future Perspectives

A qualification system was mostly classified as “mandatory” for each spine surgeon prior to performing endoscopic spine procedure, according to the survey. This might reflect that most surgeons agreed that a standard should be established for the endoscopic spine surgery to maintain a satisfactory level of procedure effectiveness and to reduce risk of complication for the patients. This could be of interest to the spine surgeon society for the qualification system to be implemented in the future.

Most respondents agreed that endoscopic spine surgery would become a gold standard in treating lumbar disc herniation. In accordance with current evidences in superiority in many outcome measures of endoscopic discectomy, including Oswestry Disability Index (ODI) score, shorter length of hospital stays, less blood loss and complications [14, 15], and endoscopic discectomy could reasonably be considered to have a potential to replace microdiscectomy as a gold standard to treat lumbar disc herniation.

There are several limitations associated with the present study. First, for a nationwide survey, this study has a relatively small sample size due to a small number of endoscopic spine surgeons in Thailand. However, we included surgeons with different ages, regions of workplace, and experiences in performing the procedure. Since most of the main authors are orthopedic surgeons, sampling bias may occur due to a small number of neurosurgeons in the study so that we could not compare different perspectives between orthopedists and neurosurgeons, which is one of the interesting aspects. The online questionnaire was required to be completed in all items or it could not be submitted and will be excluded from the study which lower the sample size and response rate. However, this let the study achieve the complete results from participants who really intended to respond to the survey. Most of the questions were in multiple-choice design that ease participants to answer quickly but may limit their response into our given choices.

## 5. Conclusions

The trend of endoscopic spine surgery has continued to grow in Thailand, shown by the rate of implementation of endoscopic spine surgery reported by Thai spine surgeons. The number of appropriate cases until one feels confident was around 28 cases. The primary motivator and obstacles were personal interest and lack of support.

## Figures and Tables

**Table 1:** The approach proportion of respondents.

Approach proportion	Number of respondents
100% interlaminar	7
90% interlaminar: 10% transforaminal	24
75% interlaminar: 25% transforaminal	9
50% interlaminar: 50% transforaminal	1
25% interlaminar: 75% transforaminal	1
100% transforaminal	0

**Table 2:** The disease proportion of respondents.

Disease proportion	Number of respondents
100% discectomy	4
90% discectomy: 10% decompression	13
75% discectomy: 25% decompression	13
50% discectomy: 50% decompression	7
25% discectomy: 75% decompression	2
10% discectomy: 90% decompression	1
100% decompression	0

**Table 3:** Endoscopic spine surgery devotion in practice and years of endoscopic experience.

	Number of respondents
*Endoscopic spine surgery devotion in practice*	
Less than 25%	18
26-25%	13
51-75%	7
More than 75%	4
*Years of endoscopic experience*	
Less than 1 year	5
1-4 years	24
5-10 years	8
More than 10 years	5

## Data Availability

The data used to support the findings of this study are available from the corresponding author upon request.
